# Incorporation of Bone Morphogenetic Protein-2 and Osteoprotegerin in 3D-Printed Ti6Al4V Scaffolds Enhances Osseointegration Under Osteoporotic Conditions

**DOI:** 10.3389/fbioe.2021.754205

**Published:** 2021-11-04

**Authors:** Xianggang Wang, Zhengyan Li, Zhonghan Wang, He Liu, Yutao Cui, Yuzhe Liu, Ming Ren, Hongsheng Zhan, Zuhao Li, Minfei Wu, Jincheng Wang

**Affiliations:** ^1^ Orthopaedic Medical Center, The Second Hospital of Jilin University, Changchun, China; ^2^ Orthopaedic Research Institute of Jilin Province, Changchun, China; ^3^ Shi’s Center of Orthopedics and Traumatology, Shuguang Hospital Affiliated to Shanghai University of TCM, Shanghai, China; ^4^ Institute of Traumatology, Shanghai Academy of TCM, Shanghai, China

**Keywords:** osteoporosis, bone tissue engineering, bone morphogenetic protein-2, osteoprotegerin, osseointegration

## Abstract

Osteoporosis is an age-related metabolic disease that results in limited bone regeneration capacity and excessive osteoclast activity. After arthroplasty in patients with osteoporosis, poor interface osseointegration resulting from insufficient bone regeneration ability often leads to catastrophic complications such as prosthesis displacement and loosening and periprosthetic fractures. In this study, we prepared a thermosensitive hydrogel loaded with bone morphogenetic protein-2 (BMP-2) to promote osteogenesis and osteoprotegerin (OPG) to inhibit excessive osteoclast activity. To construct three-dimensional (3D)-printed composite scaffolds for implantation, a hydrogel loaded with drugs was injected into porous Ti6Al4V scaffolds. The 3D-printed composite scaffolds showed good biocompatibility and sustained release of BMP-2 and OPG for more than 20 days. *In vitro* experiments indicated that composite scaffolds promoted osteogenic differentiation and reduced the osteoclastic activation simultaneously. Remarkably, immunofluorescence staining, micro-CT, histological, and biomechanical tests demonstrated that the sustained release of both BMP-2 and OPG from composite scaffolds significantly improved bone ingrowth and osseointegration in osteoporotic defects. In conclusion, this study demonstrated that the BMP-2- and OPG-loaded 3D-printed composite scaffolds can potentially promote osseointegration for osteoporotic patients after joint replacement.

## Introduction

Osteoporosis is one of the most common metabolic skeletal diseases, and it is characterized by low bone strength, limited osteogenic activity, and enhanced osteoclast resorption, thus increasing the risk of bone fractures (Ferrari, 2018; Compston et al., 2019). In bone tissue, there is a dynamic metabolic process of bone modeling and remodeling. Osteoblasts mediate the modeling process *via* secreting bone matrix and promoting calcium deposition. However, osteoclasts mediate the remodeling process *via* bone matrix resorption (Guo et al., 2021; Jia et al., 2021). However, the balance between bone formation and bone resorption in osteoporotic patients is disrupted, thus resulting in poor osseointegration after prosthesis implantation (Toepfer et al., 2021). So far, various conventional treatments have been attempted to increase the osseointegration of osteoporosis patients after joint replacement, but these treatments have not alleviated the problem (Monotti et al., 2020; Quinzi et al., 2020). Postoperative systemic administration of antiresorptive drugs is limited by various side effects and low bioavailability that consistently achieves an unsatisfactory local osseointegration effect (Frank et al., 2021). Poor osseointegration after joint replacement leads to serious complications, including prosthesis loosening or displacement and periprosthetic fracture (Zhu et al., 2021). Therefore, to ameliorate the pathological environment in the osteoporotic interface, an effective treatment that can promote osseointegration at the prosthetic interface after joint replacement is urgently required.

The titanium alloy, Ti6Al4V, is an excellent material for fabricating orthopedic implants because of its good biocompatibility, osteoconductivity, and superior corrosion resistance (Huang et al., 2021). However, the high stiffness of scaffolds causes stress shielding and poor interfacial bonding, which remains a significant problem (Abate et al., 2021). To address this challenge, 3D printing technology has been used to fabricate prostheses with a highly porous structure (Li et al., 2021). The controlled pore structure not only reduces the difference in stiffness between the prosthesis and host bone but also provides enough space for bioactive substance loading and induces bone ingrowth. Bone morphogenetic proteins (BMPs) are classic growth factors, which can induce osteogenic differentiation of stem cells *in vitro* and bone regeneration *in vivo* (Baek et al., 2021). Among all the BMPs, BMP-2 is one of the most osteogenic BMPs that can significantly promote bone induction. In the clinic, recombinant human BMP-2 (rhBMP-2) is already approved by the Food and Drug Administration (FDA) for human use (Chao et al., 2021). BMP-2 can strongly induce bone formation *via* the SMAD signaling pathway in osteoblasts (Fitzpatrick et al., 2017). In the beginning, BMP-2 will bind to the BMP receptor on the cell membrane, and then it will initiate a cascade of BMP-specific SMAD pathways, which eventually promote the expression of osteogenesis-related genes (Wang et al., 2020). In previous studies, BMP-2 was used for bone regeneration in osteoporotic defects and showed excellent results (Segredo-Morales et al., 2018; Garcia-Garcia et al., 2019). In addition, considering the special microenvironment of osteoporosis, it is necessary to block its excessive osteoclastic activity. Osteoprotegerin (OPG) is a glycoprotein belonging to the tumor necrosis factor (TNF) receptor superfamily, which is considered to be a factor that inhibits bone resorption. OPG is mainly expressed by osteoblasts; it functions to inhibit osteoclastogenesis and osteolysis *via* binding and neutralizing RANKL (Hauser et al., 2017). OPG could decrease the activity of osteoclasts by regulating the RANKL/RANK/OPG system in osteoporosis (Tu et al., 2015).

**SCHEME 1:**
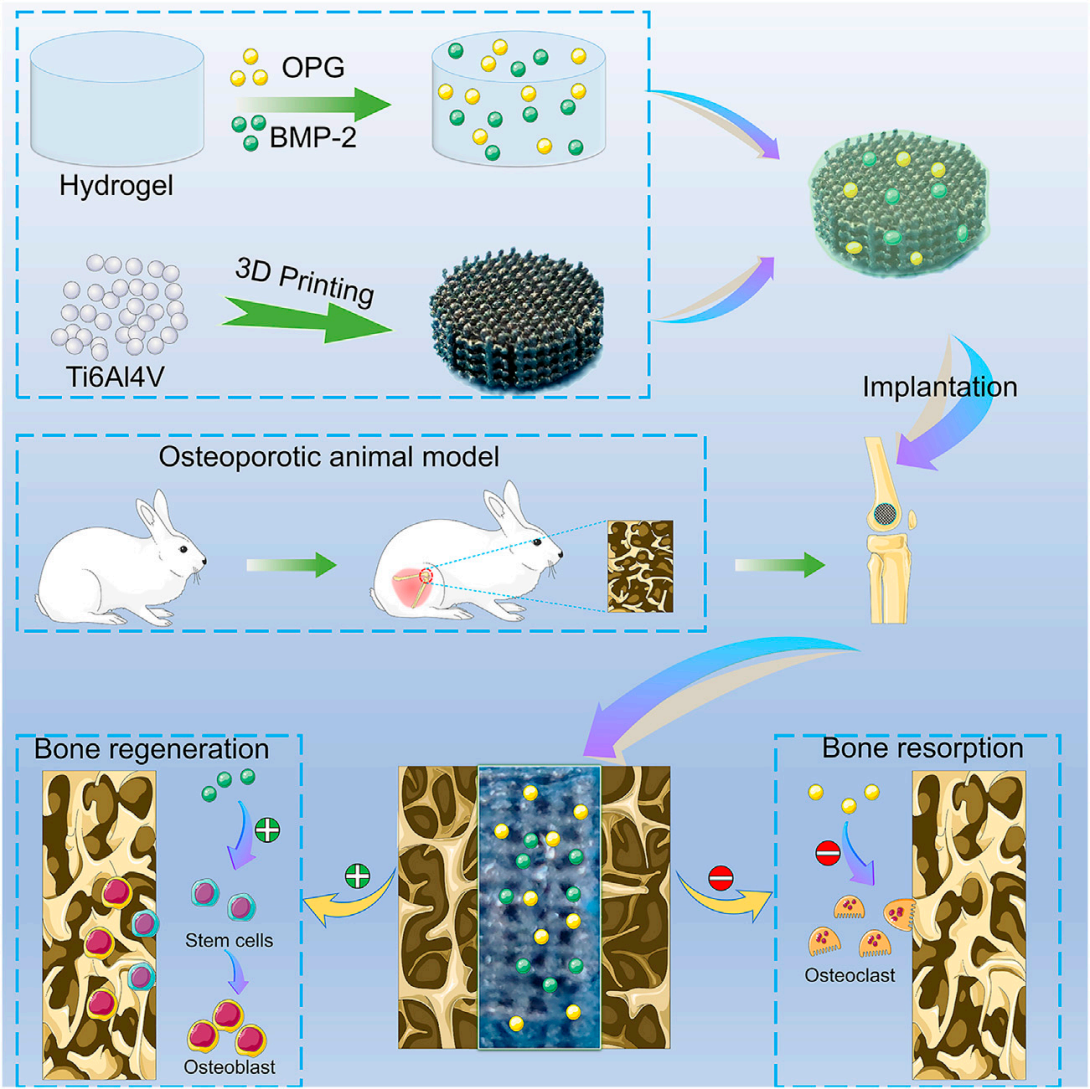
The illustration of the BMP-2/OPG-loaded 3D-printed composite scaffolds and their application as scaffolds to promote osseointegration in osteoporosis. BMP-2, bone morphogenetic protein-2; OPG, osteoprotegerin.

In this study, we prepare the thermosensitive poloxamer 407 hydrogels as a drug delivery system to incorporate BMP-2 and OPG, and the hydrogel loaded with drugs was injected into the pores of 3D-printed Ti6Al4V scaffolds to construct composite scaffolds. The Ti6Al4V scaffold with optimized pore size and porosity is designed to match the bone tissue’s mechanical strength, thus minimizing the stress shielding. We hypothesize that these BMP-2/OPG-loaded composite scaffolds will release drugs continuously and ameliorate the regenerating microenvironment in osteoporosis. Moreover, the cooperation between BMP-2 and OPG will enhance bone regeneration and inhibit bone resorption simultaneously, thus enhancing the osseointegration after the prosthesis implantation **(** Scheme 1 **)**.

## Experimental Section

### Materials

The Ti6Al4V powder was purchased from AK Medical Co., Ltd. (Beijing, China). Poloxamer 407 powder was obtained from Bayee Chemical Co., Ltd. (Hangzhou, China). BMP-2, OPG, and antibodies used in immunofluorescence were obtained from Abcam (Cambridge, UK). Low Glucose Dulbecco’s Modified Eagle’s Medium (DMEM), streptomycin double-antibody, and fetal bovine serum (FBS) were obtained from Gibco (Grand Island, NY, USA). Paraformaldehyde and phosphate-buffered saline (PBS) were obtained from Coolabar (Beijing, China). Cell Counting Kit-8 (CCK-8) and Calcein-AM/propidium iodide (PI) were supplied by Beyotime Biotechnology (Shanghai, China). The osteogenic medium used for alizarin red dye was obtained from Cyagen (Santa Clara, CA, USA). The Perfect Real-Time RE reagent kit from Takara Bio (Dalian, China), 2× Fast SYBR Green Master Mix from Roche Diagnostics (Basel, Switzerland), and TRIzol reagent (Invitrogen, CA, USA) were used. The Runx-2, OPN, and RANKL antibodies were supplied by Abcam (Cambridge, UK) and DAPI from Solarbio (Beijing, China). The Electrochemical Immunoassay Kit was obtained from Roche-Mannheim (Mannheim, Germany). The tartrate-resistant acid phosphatase (TRAP) staining kit was obtained from Sigma-Aldrich (St. Louis, MO, USA). The ultrapure water used in the study was obtained from a Milli-QA10 filtration system (Millipore, Billerica, MA, USA).

### Preparation of Bone Morphogenetic Protein-2/Osteoprotegerin-Loaded 3D-Printed Composite Scaffolds

#### Fabrication of the 3D-Printed Ti6Al4V Scaffolds

The 3D-printed porous Ti6Al4V scaffolds were fabricated as previously described (Bai H. et al., 2020). Briefly, a 3D model of the disk-shaped scaffold (Φ6 mm × L 3 mm) was established with the following parameters: pore size = 800 μm, porosity = 70%, and strut size = 300 µm. Spherical pre-alloyed medical-grade Ti6Al4V powder (Grade 23, particle size 45–100 µm) was used to fabricate the porous scaffold using the electron beam melting (EBM) machine (Q10, Arcam, Gothenburg, Sweden). To verify whether the parameters of the prepared scaffolds were consistent with the predesign, a scanning electron microscope (SEM; JSM-6700F, JEOL, Japan) and Micro-CT scanner (SkyScan 1076, Kontich, Belgium) were used to evaluate the pore size and porosity. All obtained scaffolds were ultrasonically cleaned and washed sequentially in acetone, ethanol, and deionized water for 10–15 min in each solution.

#### Preparation of the Hydrogel

The hydrogel was prepared by mixing the powder with 0.01 M of PBS (pH = 7.4, 4°C) at the ratio of 25%:75% (w/w) at 4°C overnight. The rheological properties of the poloxamer 407 hydrogels were assessed on a rheometer (Discovery HR-2, TA Instruments, New Castle, DE, USA) from a Peltier plate. The dynamic mechanical data of the storage modulus (*G′*) and viscous modulus (*G″*) of the hydrogels were tested at different temperatures. Moreover, to observe the morphology of the hydrogel and scaffolds, the SEM was also used at 3-kV acceleration voltage.

#### Construction of 3D-Printed Composite Scaffolds

After poloxamer 407 was completely dissolved, a homogeneous and transparent solution was obtained. Then, the BMP-2 solution (7.5 μg/ml, in 0.01 M of PBS, pH = 7.4, 4°C) and the OPG solution (0.1 mg/ml, in 0.01 M of PBS, pH = 7.4, 4°C) were added to the prepared hydrogel solution. To prepare the drug-loaded composite scaffolds, the porous Ti6Al4V scaffolds were inserted into a 96-well plate, and the hydrogel containing BMP-2 and OPG was injected into the porous Ti6Al4V scaffolds at 4°C. Finally, the composite scaffolds within the 96-well plate were warmed to 37°C in an incubator to gelate the hydrogel solution.

#### Bioactive Bone Morphogenetic Protein-2 and Osteoprotegerin Release Profile

To investigate the bioactive release of BMP-2 and OPG *in vitro*, the BMP-2/OPG-loaded 3D-printed composite scaffolds were put in a 24-well culture plate. It was soaked in PBS at 37°C. Medium measuring 2 ml was collected and changed at predetermined time points. BMP-2 and OPG release levels were tested using the BMP-2 and OPG ELISA Kits and analyzed by a microplate reader (Multiskan EX, Thermo Fisher Scientific, Shanghai, China).

### *In Vitro* Cell Experiments

#### Isolation and Culture of Osteoporosis-Derived Bone Marrow Mesenchymal Stem Cells

The osteoporosis-derived bone marrow mesenchymal stem cells (OP-BMSCs) were cultured and isolated as previously described (Zhao et al., 2020). Briefly, the OP-BMSCs were obtained from the long bone marrow of female New Zealand rabbits 10 months after ovariectomy (OVX). OP-BMSCs were cultured in a low-glucose DMEM containing 1% streptomycin–penicillin and 10% FBS in a humidified incubator at 37°C and 5% CO_2_. Cells were digested and passaged at approximately 80% confluence. The third passage of cells was used for *in vitro* experiments.

#### Biocompatibility

The CCK-8 experiment was used to investigate the cell proliferation of composite scaffolds. OP-BMSCs with a density of 1 × 10^4^ cells/well in 24-well culture plates were seeded in porous scaffolds (S), hydrogel-incorporated porous scaffolds (SH), BMP-2-loaded hydrogel-incorporated porous scaffolds (SH/BMP-2), OPG-loaded hydrogel-incorporated porous scaffolds (SH/OPG), and dual BMP-2 and OPG-loaded hydrogel-incorporated porous scaffolds (SH/Dual). The cell proliferation was tested on days 1, 4, and 7. At every time point, the reaction solution was added to each well for 2.5 h of incubation at 37°C. The optical density (OD) was measured using a microplate reader at 450 nm. Calcein-AM/PI staining was performed after 3 days of culture according to the manufacturer’s protocol to determine the cell viability in each group. Briefly, 2 μM of Calcein-AM and 4.5 μM of PI were added to the samples, incubated for 15 min at 37°C in the dark, and then evaluated under a confocal laser scanning microscope (CLSM; FV1000, Olympus, Tokyo, Japan).

#### Alizarin Red S Staining

The OP-BMSCs were seeded on the S, SH, SH/BMP-2, SH/OPG, and SH/Dual groups at a density of 1 × 10^5^ cells/well in a 24-well plate. After the cells adhered, the medium was changed to an osteogenic induction medium. The medium was changed every 3 days. The osteogenic differentiation ability was evaluated at 14 and 21 days. At the scheduled time point, cells were fixed with 4% paraformaldehyde for 30 min at 37°C and washed three times with PBS. The ARS solution was added to the samples at room temperature for 20 min. After being washed with PBS to remove the residual stains, the images were taken using a microscope (Olympus Ⅸ). To quantify the results of ARS, samples were dissolved with 10% cetylpyridinium chloride, and the coloration of each sample was read by a microplate reader at 562 nm.

#### Real-Time Quantitative PCR

After osteogenic induction culture of OP-BMSCs (1 × 10^5^ cells/well) in 24-well plates for 14 and 21 days, the expression of runt-related transcription factor-2 (*Runx-2*), osteopontin (*OPN*), and receptor activator of NF-κB ligand (*RANKL*) were investigated using RT-qPCR. Total RNA was collected using TRIzol reagent. The purity of RNA was evaluated by a NanoDrop 2,000c Spectrophotometer (Thermo Fisher Scientific, Waltham, MA, USA). Only an A_260_/A_280_ value around 2.0 was accepted for further analysis. Synthesis of cDNA was performed in a 20-µl reaction volume *via* a Perfect Real-Time RE reagent kit. Then, the qPCR amplification and detection were analyzed using 2 × Fast SYBR Green Master Mix on a LightCycler 480 (Bio-Rad CFX Manager 3.1, Hercules, CA, USA). Based on the gene expression ratio among different groups, the relative expression level of mRNA was normalized to GAPDH and calculated using the 2^−ΔΔct^ method. Primer sequences are listed in Supplementary Table S1.

### *In Vivo* Osseointegration of 3D-Printed Composite Scaffolds

#### Preparation of Osteoporotic Animal Models

Animal experiments were conducted in compliance with the National Institutes of Health’s Guide for the Care and Use of Laboratory Animals (NIH Publications No. 8023, revised 1978). All experimental protocols for animals were approved by the Animal Care and Use Ethics Committee at Jilin University. The osteoporotic rabbit models were prepared by OVX according to our previous studies (Bai H. et al., 2020). Briefly, a total of 46 female New Zealand rabbits were randomly divided into two groups; namely, 43 of them underwent bilateral OVX surgery, and three of them received sham surgery. Ten months later, their serum estrogen levels were tested using an Electrochemical Immunoassay Kit. Three rabbits of each group were sacrificed, and their distal femurs were used for Micro-CT measurement and H&E staining to confirm the osteoporosis status.

#### Implantation of 3D-Printed Composite Scaffolds

Forty residual osteoporotic rabbits were enrolled in the osseointegration experiments, and they were randomly divided into five groups: S, SH, SH/BMP-2, SH/OPG, and SH/Dual. Briefly, the osteoporotic rabbits were anesthetized using 3% (w/v) pentobarbital (50 mg/kg). After the skin preparation and sterilization, a longitudinal incision at the distal femur site was made to expose the lateral condyle. Then, a cylindrical bone defect (6.0 mm in diameter and 3.0 mm in depth) was created with a bone drill. 3D-printed composite scaffolds were implanted into each animal, and incisions were closed subsequently in layers by absorbable sutures. After the operation, all rabbits were allowed free movement. Penicillin (40,000 U) was intramuscularly injected for 3 days to prevent infection postoperatively. Three months after the implantation, all rabbits were sacrificed by overdose with 3% (w/v) pentobarbital (150 mg/kg), and the bilateral femurs were harvested for subsequent detection.

#### Micro-CT Analysis

The specimens were scanned using Micro-CT (90 kV, 114 mA, 18-µm image pixel size) to evaluate the effect of bone regeneration and osseointegration. A cylinder (6.0-mm diameter and 3.0-mm height) was selected as the region of interest (ROI). The 3D reconstruction was performed by multimodal 3D visualization software (NRecon 1.7.1.0 software, Kontich, Belgium). The quantitative analysis of the ROI was conducted by Micro-CT auxiliary software (VG studio Volume Graphics GmbH, Heidelberg, Germany), including bone volume/tissue volume ratio (BV/TV, %), trabecular thickness (Tb.Th, mm), trabecular separation (Tb.Sp, mm), and trabecular number (Tb.N, 1/mm).

#### Immunofluorescence Staining

As previously reported, the immunofluorescence staining of osteogenesis-related genes at the regenerated bone site was carried out (Zhao et al., 2020). Briefly, bone tissue sections were incubated with Runx-2, OPN, and RANKL antibodies overnight at 4°C. After that, the specimens were washed three times using PBS, and cell nuclei were stained with DAPI. The positively stained samples were imaged with a CLSM; the intensity of fluorescence was quantitatively analyzed by image Pro-Plus (IIP) 6.0 (National Institutes of Health, Bethesda, MD, USA).

#### Push-Out Test

A standard push-out test was performed to evaluate the integration strength of the bone-scaffold interface in each group. The specimens were placed on the plate for a detaching test (1.0 mm/min) using a closed-loop servo-hydraulic testing machine (MTS MiniBionix, Minneapolis, MN, USA). The maximum pushing force was recorded when scaffolds moved away from the bone.

### Statistical Analysis

All data were represented as the mean ± SD from at least three independent experiments. The statistical analyses were performed *via* Student’s t-test or one-way ANOVA, followed by the least significant difference test for multiple comparisons using SPSS 19.0 software (SPSS Inc., Chicago, IL, USA). *p* < 0.05 was deemed to indicate statistical significance.

## Results and Discussion

### Preparation and Characterization of 3D-Printed Composite Scaffolds

**FIGURE 1:**
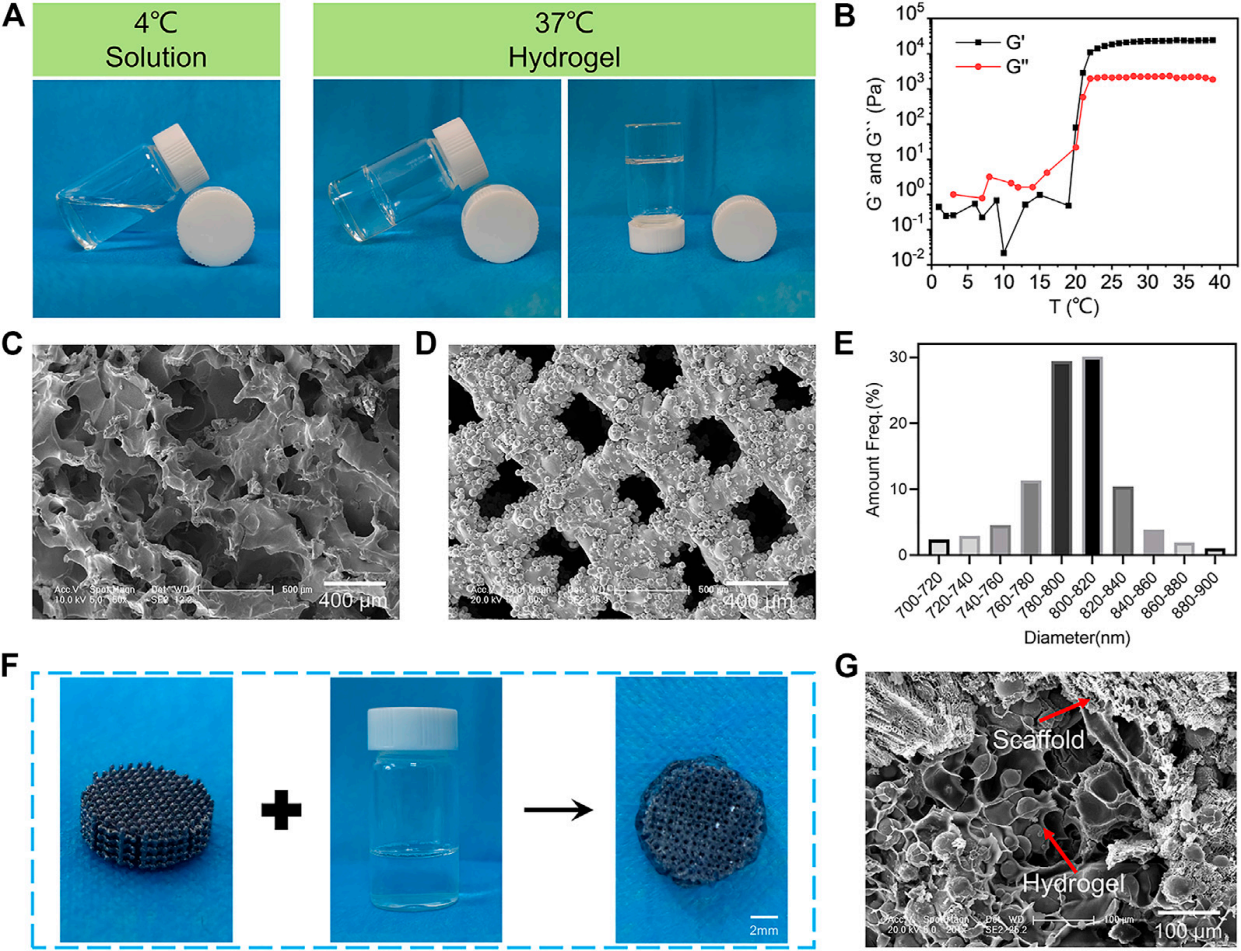
Characterization of the BMP-2/OPG-loaded 3D-printed composite scaffolds. **(A)** The thermosensitive property of the poloxamer 407 hydrogel. **(B)** The rheological analysis of the storage modulus (*G′*) and loss modulus (*G″*) of the hydrogel as a function of temperature. **(C)** Morphologies of the hydrogels observed by SEM. **(D)** Morphologies of the 3D-printed porous Ti6Al4V scaffolds. **(E)** The pore size distribution of 3D-printed porous Ti6Al4V scaffolds. **(F)** Illustration demonstrating the hydrogel loaded with drugs injected into porous scaffolds to prepare the BMP-2/OPG-loaded 3D-printed composite scaffolds. **(G)** SEM image of the 3D-printed composite scaffolds. BMP-2, bone morphogenetic protein-2; OPG, osteoprotegerin; SEM, scanning electron microscopy.

The poloxamer 407 hydrogel is widely used in bone tissue engineering because of its reversible thermo-responsive properties, superior drug delivery capabilities, excellent biocompatibility, and biodegradability (Yang et al., 2020). In this study, thermosensitive poloxamer 407 hydrogels were successfully prepared (Figure 1A). The poloxamer 407 solution was liquid at 4°C, and it was converted to a gel state when the temperature rose to 37°C. Furthermore, a rheological analysis of the thermosensitive hydrogel confirmed that both the storage modulus (G*′*) and loss modulus (G*″*) of the hydrogel rapidly increased after 21°C, suggesting that the temperature of the hydrogel to sol-gel transition was about 21°C (Figure 1B). SEM observations showed that the hydrogel had a porous internal structure with a pore size of about 100–200 µm (Figure 1C). The thermosensitive property of the poloxamer 407 hydrogel made it suitable for bone tissue engineering because the normal human body temperature is 37°C.

In addition, the 3D-printed porous Ti6Al4V scaffolds were successfully fabricated using the EBM machine. SEM images of the porous scaffolds revealed the interconnected pore structure (Figure 1D). A quantitative analysis (using ImageJ software) showed that the diameter of micropores ranged from 760 to 840 μm (Figure 1E), and the average pore size was 791.97 ± 11.32 μm. Subsequently, the porosity of the prepared porous Ti6Al4V scaffolds was determined by Micro-CT. The results revealed that the porosity of the scaffolds was 70.13% ± 2.43%. Therefore, the actual pore size and porosity of the printed porous Ti6Al4V scaffolds were consistent with the predesign model (800 μm and 70%, respectively). The osseointegration ability of the porous prosthesis after implantation is closely related to the porosity and pore size. For porous implants, the porosity should be above 50%, specifically in the range of 65%–75%. Its structure and mechanics are bionic with human trabecular bone, which is conducive to osseointegration. As for the pore size, previous research has revealed that when the pore is greater than 300 μm, it is beneficial for nutrients and oxygen to penetrate the interconnected micropores (Chen et al., 2020). Furthermore, studies on the relationship between micropore diameter and bone regeneration have indicated that a pore size between 600 and 800 μm is beneficial for osseointegration in osteoporosis (Bai H. et al., 2020; Qiao et al., 2020). Therefore, the pore size and porosity of our prepared porous scaffolds were 791.97 μm and 70.13%, respectively, which are ideal for osseointegration in osteoporosis.

The synthesis procedures for BMP-2/OPG-loaded 3D-printed composite scaffolds are depicted in Figure 1F. At 4°C, the porous Ti6Al4V scaffold was soaked in the BMP-2/OPG-loaded poloxamer 407 solutions to fill the scaffold pores uniformly. At 37°C, the solution turned into a hydrogel, and the hydrogel and the scaffold were combined into a complex. The SEM images also confirmed that the scaffold pores were filled with hydrogels (Figure 1G). These results indicated that the BMP-2/OPG-loaded 3D-printed composite scaffolds were prepared successfully.

Hydrogels are always used for drug delivery systems to release the drugs in a controlled manner (Borges et al., 2021). The reversible thermo-responsive property of poloxamer 407 hydrogel allows it to undergo gelation near body temperature (approximately 37°C) and remain a continuous drug delivery device *in vivo* (Beard et al., 2021b). At lower temperatures, poloxamer 407 exists in the form of a solution, during which it can be loaded with therapeutics for later release from its gel state (Beard et al., 2021a). A previous study has demonstrated that poloxamer 407 hydrogel loaded with drugs can be successfully injected into porous scaffolds and achieve sustained release (Bai H. T. et al., 2020).

### The Release Profiles of the 3D-Printed Composite Scaffolds

**FIGURE 2:**
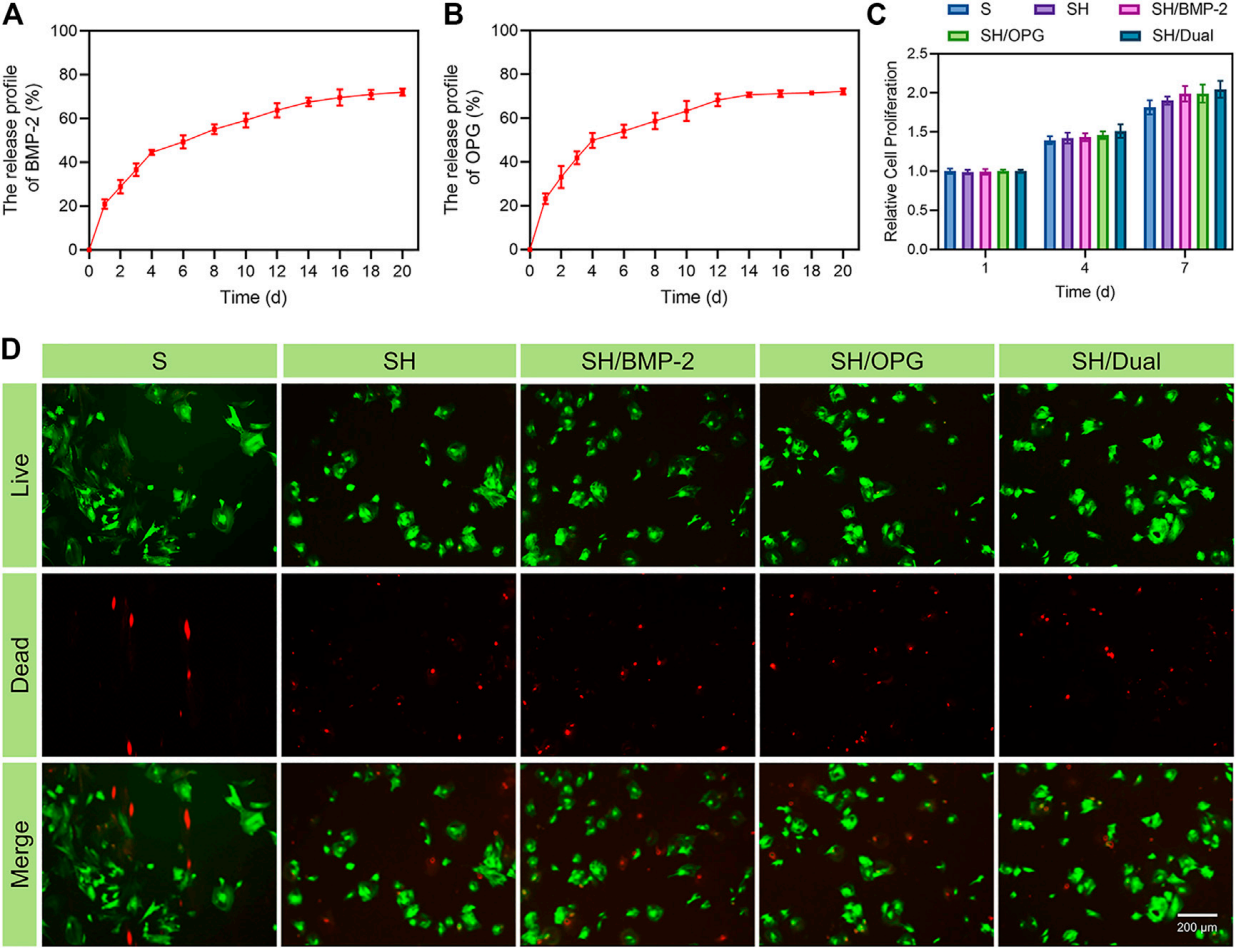
The release profiles and biocompatibility of the 3D-printed composite scaffolds. **(A)** The release profile of BMP-2. **(B)** The release profile of OPG. **(C)** The cell proliferation of BMSCs in different groups. **(D)** Calcein-AM/PI staining of BMSCs at day 3 (green represents live cells, whereas red represents dead cells). BMP-2, bone morphogenetic protein-2; OPG, osteoprotegerin; BMSCs, bone marrow mesenchymal stem cells; PI, propidium iodide.

For a drug delivery system, sustained release is critical to achieving the intended therapeutic effect. The release profiles of the BMP-2/OPG-loaded 3D-printed composite scaffolds indicated that the slow degradation of hydrogels allowed the BMP-2 and OPG to be delivered in a controlled manner. Figures 2A,B show that the kinetic release of BMP-2 and OPG was recorded in 20 days. Initially, there was a rapid release of drugs in the first 4 days, especially on day 1. For BMP-2, the release rate was about 20.9% ± 1.1% at day 1. On day 4, the percentage of the released drug was approximately 44.5% ± 2.3%. From day 4 to day 16, the release rate slowed down; and on day 16, the total fraction of drug release was 69.6% ± 1.3%. After that, the release profile increased slowly. As for OPG, the release rate was about 23.1% ± 2.3% on day 1 and approximately 49.9% ± 2.7% on day 4. Similar to BMP-2, the release rate slowed down from day 4 to day 16, with a total fraction of drug release of 61.1% ± 1.2% at day 16. Because of protein denaturation and low initial drug concentration, after 20 days, the residual drug content was very low. The residual drug concentration was difficult to measure accurately; that is why only 70% of the drugs were detected. The release of drugs in the hydrogel was the result of drug diffusion and hydrogel degradation. At the early time point, the rapid drug release mainly resulted from the drug diffusion since there was no significant hydrogel degradation. Moreover, because of the porous structure of both hydrogels and scaffolds, there was a broad hydrogel–liquid interface (Colucci et al., 2021). Through the diffusion of drugs on the surface, the broad interface was initially mainly responsible for rapid release, which was acceptable in the clinic and consistent with drug delivery patterns in other studies (Katakam et al., 2019; Tundisi et al., 2021). Later, the degradation rate was influenced by the drug diffusion and hydrogel degradation and the interlink of chemical bonds and intermolecular hydrogen bonds between drugs and the poloxamer 407 (Borges et al., 2021). Thus, the sustained release of drugs was achieved (Solanki et al., 2019). Because the protein concentration is impossible to detect *in vivo*, *in vitro* experiments simulated the *in vivo* release pattern. *In vitro*, we studied the release curves of growth factors in composite scaffolds in PBS. PBS maintains a constant pH, and the osmotic pressure and ion concentration are similar to those of the human body, and they simulate the effect of body fluid. Therefore, it is reasonable to believe that the growth factors in the composite scaffolds can obtain a similar release curve *in vivo*. This degradation mode will meet the clinical requirements, and the initial rapid release is conducive to the drug reaching a high concentration at the defect and playing a rapid therapeutic role while alleviating the disease. The slow release in the later stage is beneficial to maintaining the drug concentration (Santimetaneedol et al., 2019). These results indicate that the poloxamer 407 hydrogel was an excellent drug delivery system for sustained release in bone regeneration.

### The Biocompatibility of the 3D-Printed Composite Scaffolds

In addition to sustained drug release, the biocompatibility of the 3D-printed composite system is the basis for biological applications. To test the biocompatibility of the BMP-2/OPG-loaded 3D-printed composite scaffolds, a CCK-8 assay was conducted to analyze its effect on cell proliferation. OP-BMSCs were seeded on 3D-printed composite scaffolds in different groups for 1, 4, and 7 days. Figure 2C indicates that cells proliferated gradually in each group within 7 days. There was no significant difference between the groups, but the drug-loaded scaffold groups showed an increased cell proliferation rate on day 7. Previous studies reported that BMP-2 and OPG were mainly responsible for cell differentiation, with little effect on cell proliferation (Yarygin et al., 2020; Wang et al., 2021; Zhang et al., 2021). In addition, the images of Calcein-AM/PI staining indicated that the OP-BMSCs had good cell viability in all groups on day 3 (Figure 2D). These results indicated that the incorporated drugs, hydrogels, and scaffolds used in this study had no cytotoxicity, suggesting that the BMP-2/OPG-loaded 3D-printed composite scaffolds had good biocompatibility and were suitable for *in vivo* applications.

### Osteogenic and Osteoclastic Differentiation on the 3D-Printed Composite Scaffolds

**FIGURE 3:**
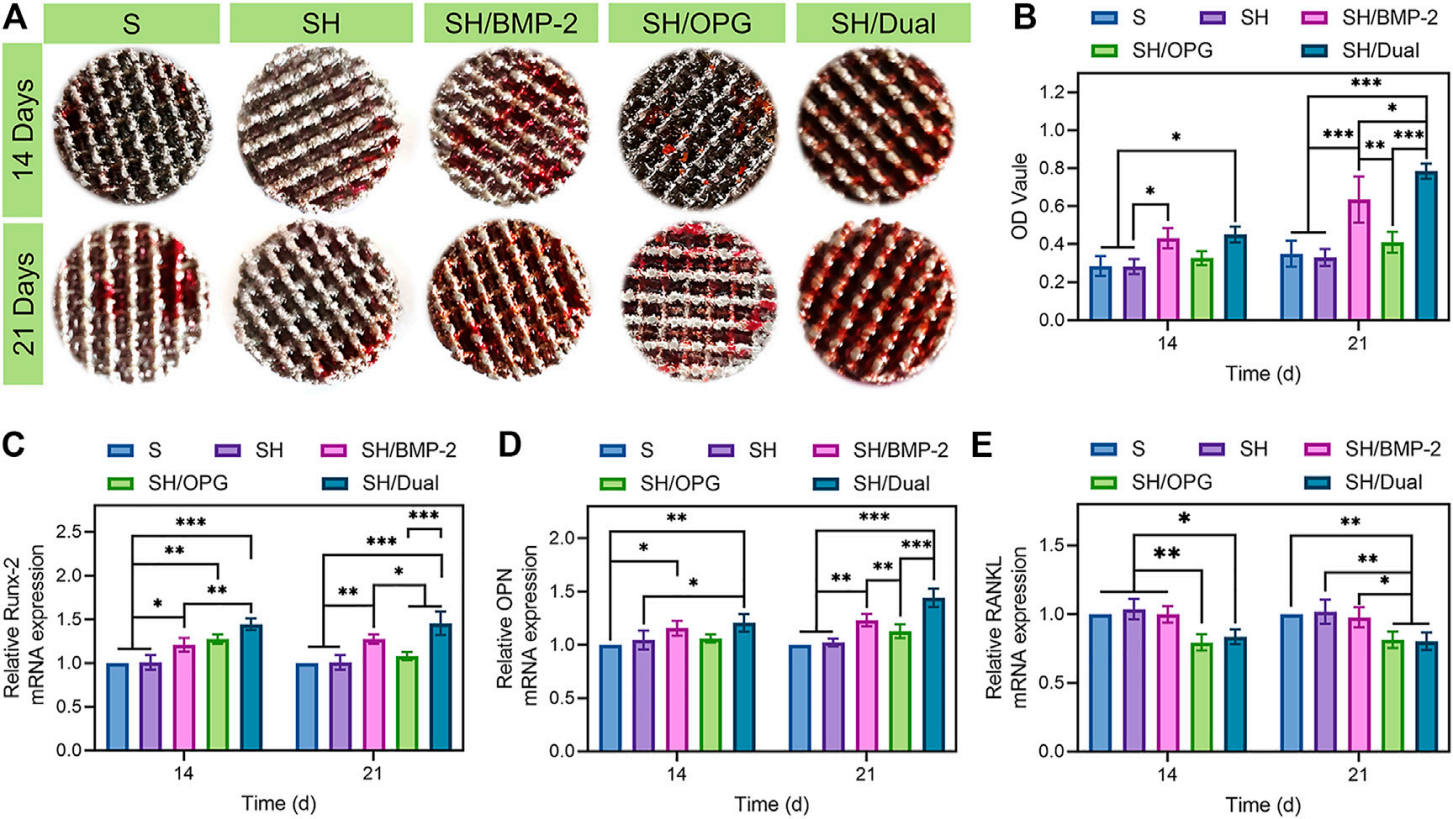
The osteogenic and osteoclastic activity of BMSCs on different 3D-printed composite scaffolds. **(A)** The alizarin red staining of the 3D-printed composite scaffolds in different groups. **(B)** Quantitative analysis of the ARS results. **(C–E)** RT-qPCR analyses of the gene expressions of *Runx-2*, *OPN*, and *RANKL*. * indicates significant difference between groups, *p < 0.05, **p < 0.01, and ***p < 0.001. BMSCs, bone marrow mesenchymal stem cells.

In addition to cell proliferation, osteogenic differentiation of BMSCs is an important event for the initiation of bone regeneration. ARS attaining, which indicated the number of calcified deposits in BMSCs (Queiroz et al., 2021), was used to evaluate the osteogenic effects of the 3D-printed composite scaffolds on OP-BMSCs. Gross images revealed that calcified nodules were increased from 14 to 21 days in each group (Figure 3A), but there was no significant difference among the S, SH, and SH/OPG groups at each time point. Moreover, OPG did not have significant effects on calcium deposition. As predicted, the BMP-2-loaded groups had significantly more calcified nodules than the S and SH groups at both time points (Figure 3B). The difference in nodules increased as time went on (*p* < 0.05 at day 14 and *p* < 0.001 at day 21). More importantly, we observed more calcium deposition in the SH/Dual group compared with the SH/BMP-2 group (*p* < 0.05). These results demonstrated that compared with BMP-2 alone, although OPG did not significantly affect osteogenesis, the cooperation of BMP-2 and OPG promoted mineralized matrix formation more significantly.

To further analyze the effect of the BMP-2/OPG-loaded 3D-printed composite scaffolds on osteogenic differentiation and osteoclastic differentiation, the expression of related genes, including Runx-2, OPN, and RANKL, were evaluated *via* RT-qPCR. Typically, Runx-2 is one of the transcription factors in the Runx family (Song et al., 2020). It is considered an early-stage marker that indicates the differentiation of osteoblasts (Zahid and Ghafoor, 2021). Figure 3C shows that on both days 14 and 21, the expression of *Runx-2* was significantly higher in SH/BMP-2 than in the S and SH groups. More importantly, the expressions of *Runx-2* in SH/Dual were significantly higher than in SH/BMP-2 in both time points. This means that the existence of OPG can more significantly improve bone regeneration compared with BMP-2 alone. OPN is a glycophosphoprotein in the extracellular matrix that plays an essential role during osteoblastic differentiation (Han et al., 2021). From Figure 3D, the activity of *OPN* was significantly upregulated in BMP-2-loaded composite scaffolds on day 14 and day 21. However, though there was no significant difference between the SH/Dual and SH/BMP-2 groups, and the SH/Dual group showed higher expression of *OPN* than did the other groups. Figures 3B–D clearly show a similar trend of osteogenesis between the SH/Dual group and the SH/BMP-2 group. At the early time point, they had a similar effect on bone regeneration, but as time went on, the expression levels of osteogenic differentiation markers tended to be higher in the SH/Dual group. This phenomenon may result from BMP-2 inducing the osteoblastic differentiation of BMSCs at the early time point. Lately, the existence of OPG inhibited the osteoclast-related differentiation, which maintains the osteogenic environment.

Under osteoporotic conditions, adipogenesis and osteoclastogenesis are enhanced while osteogenesis is inhibited (Cosman et al., 2014). For BMSCs (well-known precursor cells for adipocytes and osteoblasts), under osteoporotic conditions, adipogenesis and osteoclastogenesis overwhelm osteogenesis (Shen et al., 2018). The osteoclastogenesis process is achieved through RANKL/OPG/RANK pathway (Lai et al., 2020) and osteoclastogenic cytokines including interleukin-6 (IL-6), TNF-α, and macrophage colony-stimulating factor (M-CSF) (Huang et al., 2020). RANKL, secreted by osteoblasts, can induce osteoclast function through binding to RANK on the surface of osteoclasts (Zhang et al., 2015). In experiments performed *in vitro*, the expression levels of RANKL in OP-BMSCs are often used to detect the effects of osteoclastogenesis (Bai H. T. et al., 2020). In this study, further evidence was offered by measuring the expression of osteoclast-related genes to make this point clearer. The *RANKL* gene can regulate the differentiation and function of osteoclasts by binding to RANK, which is located on the osteoclast membrane (Cang et al., 2021). The activation of RANK promotes osteoclastic effects and increases bone resorption (Liu et al., 2021). Therefore, the lower expression level of RANKL reveals a lower level of osteoclastic differentiation. In Figure 3E, both the SH/OPG and SH/Dual groups showed reduced levels of RANKL expression than did the other three groups. All these statistical differences indicated that the cooperation of BMP-2 and OPG significantly improved osteogenic differentiation and inhibited osteoclastic activation. Therefore, it is predictable that the dual BMP-2/OPG-loaded 3D-printed composite scaffolds will regulate the osteoporotic microenvironment to promote osteogenesis and inhibit osteolysis, thus promoting osseointegration after joint replacement.

### Validation of the Ovariectomy Rabbit Models

All the female rabbits were kept alive, and no infection or other surgical complications occurred throughout the experiment. In osteoporotic animals, the balance between bone resorption and bone regeneration is broken. This means that enhanced bone resorption will cause decreased BMD, which will increase the risk of fracture. As previously demonstrated, the OVX rabbits will have a deficiency of estrogen levels, and low estrogen concentration can inhibit osteoblast function and promote bone resorption (Permuy et al., 2019). In this way, the osteoporotic animals were established by OVX. To verify the establishment of the osteoporotic model, six rabbits were sacrificed 10 months after the OVX surgery, including three OVX and three sham rabbits. From Supplementary Figure S1, the H&E staining of distal femur slices indicated that the trabecular structure in the OVX group was much thinner and looser than in the sham groups. In addition, the serum estrogen level in non-OVX rabbits was 3.2-fold higher than in OVX rabbits (Supplementary Figure S2). The statistical analyses also demonstrated that BV/TV (Supplementary Figure S3) and BMD (Supplementary Figure S4) in OVX rabbits were significantly decreased compared with those in the sham group (*p* < 0.05). In summary, all data collectively confirmed that the osteoporotic rabbits were successfully established 10 months after OVX surgery.

### Osseointegration of the 3D-Printed Composite Scaffolds in Osteoporosis

**FIGURE 4:**
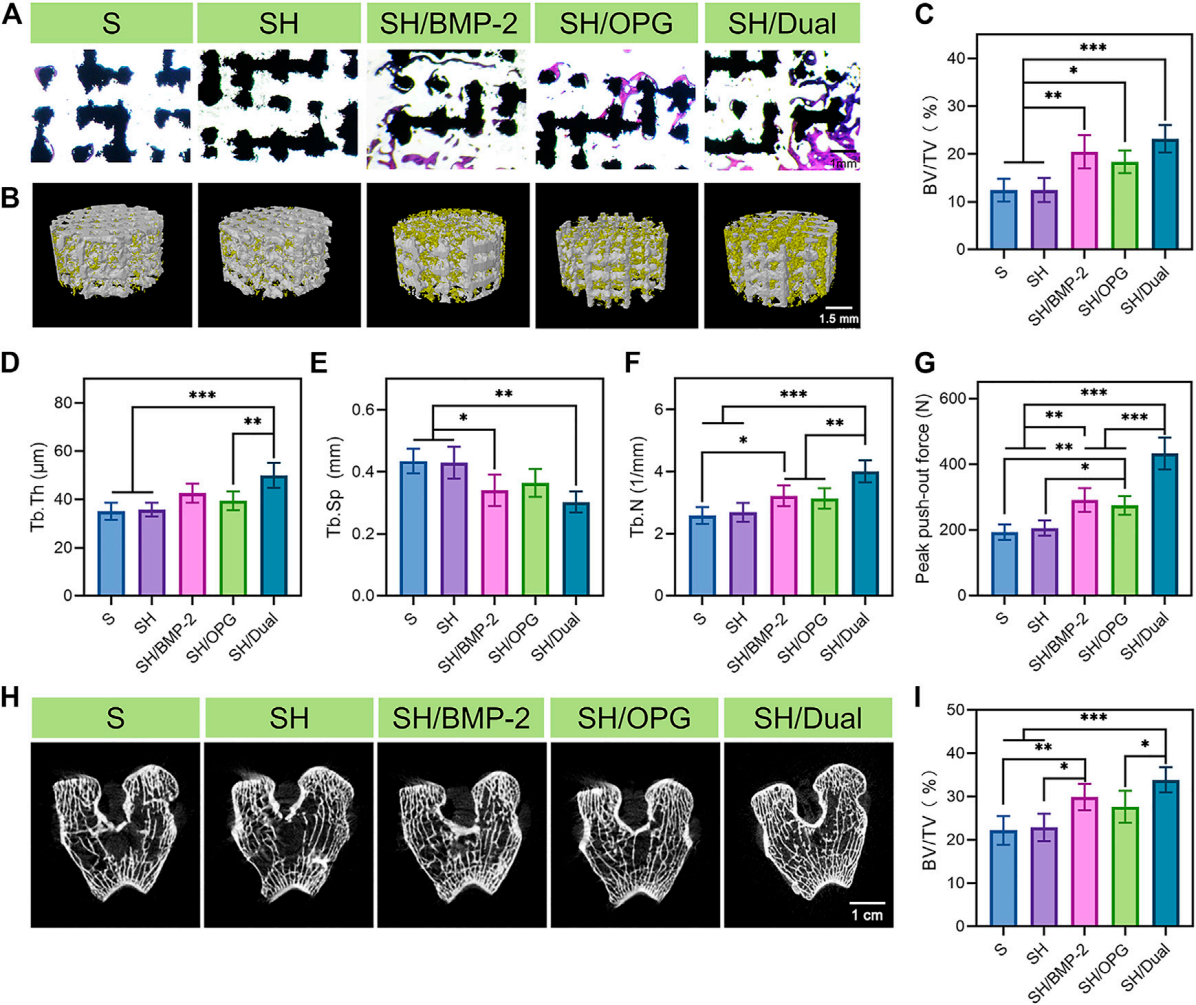
Bone regeneration and osseointegration in the 3D-printed composite scaffolds. **(A)** Masson’s trichrome staining of the regenerated bone tissue in and around the 3D-printed composite scaffolds. **(B)** 3D reconstruction images of different groups (the yellow substance represents the new bone tissue). **(C–F)** Micro-CT analyses of BV/TV, Tb.Th, Tb.Sp, and Tb.N in each group. **(G)** The analysis of the push-out test. **(H)** The CT images of the distal end of the implants in each group. **(I)** Quantitative analysis of the 2-cm-long femur at the distal end in each group. * indicates significant difference between groups, *p < 0.05, **p < 0.01, and ***p < 0.001. BV/TV, bone volume/tissue volume ratio; Tb.Th, trabecular thickness; Tb.Sp, trabecular separation; Tb.N, trabecular number.

After validating the osteoporotic animal models, the OVX rabbits were used to evaluate the osseointegration efficiency of BMP-2/OPG-loaded 3D-printed composite scaffolds. The composite scaffolds were successfully implanted into the defects at the distal femur in the osteoporotic rabbits. Masson’s trichrome staining was performed 3 months after implantation to observe the bone ingrowth and bone interface bonding. From Figure 4A, it was found that there was more bone ingrowth in the BMP-2- and OPG-loaded groups, while less was observed in the S and SH groups. In addition, much more bone regeneration was revealed in the SH/Dual group compared with the SH/BMP-2 and SH/OPG groups. Moreover, Micro-CT was employed to evaluate new bone formation in the implanted site. The 3D reconstruction images of the porous scaffolds are shown in Figure 4B, where the yellow part represents the regenerated bone tissue and the white part indicates the porous scaffolds. It was clear to see more bone tissue on the surface and in the pores in the SH/Dual group and moderate tissue in the SH/BMP-2 and SH/OPG groups. However, limited bone formation was found in the S and SH groups. Quantitative morphological results of Micro-CT were further analyzed and depicted in Figures 4C–F. The BV/TV values of S, SH, SH/BMP-2, SH/OPG, and SH/Dual were 12.41% ± 0.93%, 12.47% ± 2.50%, 20.45% ± 3.84%, 18.39% ± 2.63%, and 23.18% ± 2.84%, respectively. Furthermore, the results of Tb.Th, Tb.Sp, and Tb.N also indicated that BMP-2 and OPG could significantly enhance bone mass and quality in the defects. Taken together, the histological and micro-CT results revealed that the BMP-2/OPG-loaded 3D-printed composite scaffolds could promote bone regeneration and bone ingrowth after porous prosthesis implantation.

The probability of prosthesis displacement and loosening and periprosthetic fracture can be predicted by evaluating the osseointegration strength of the interface and the surrounding bone mass (Kang et al., 2018; Cai et al., 2020). The push-out test was conducted to evaluate the osseointegration strength of the interface between the implants and surrounding bone (Figure 4G). The maximum push-out force values of the SH/Dual group were 1.5-fold and 1.6-fold higher than those of the SH/BMP-2 and SH/OPG groups (*p* < 0.001), and 2.2-fold and 2.1-fold higher than those of the S and SH groups (*p* < 0.001), respectively. It is well known that some severe complications, including prosthesis displacement and loosening, are because of the weak bone integration of the prosthesis and surrounding bone. Therefore, the dual existence of BMP-2 and OPG can significantly reduce the risk of implant loosening and displacement. In addition, the occurrence of periprosthetic fracture is often related to the bone mass around the prosthesis after joint replacement. In this study, we performed a micro-CT scan and quantitative analysis. The CT images of the distal end of the implants indicated that the SH/Dual group had the densest trabecular bone structure among the groups (Figure 4H). Quantitative analysis of the 2-cm-long femur at the distal end of the femurs where the implants were located revealed that the BV/TV values of S, SH, SH/BMP-2, SH/OPG, and SH/Dual were 22.21% ± 2.98%, 22.91% ± 2.83%, 29.89% ± 2.71%, 27.66% ± 3.30%, and 33.88 ± 2.61%, respectively (Figure 4I). These results indicated that BMP-2/OPG-loaded 3D-printed composite scaffolds could improve the bone mass around the implants, which can be expected to reduce the occurrence of periprosthetic fractures.

### Bone Formation and Resorption Markers on the Interface

**FIGURE 5:**
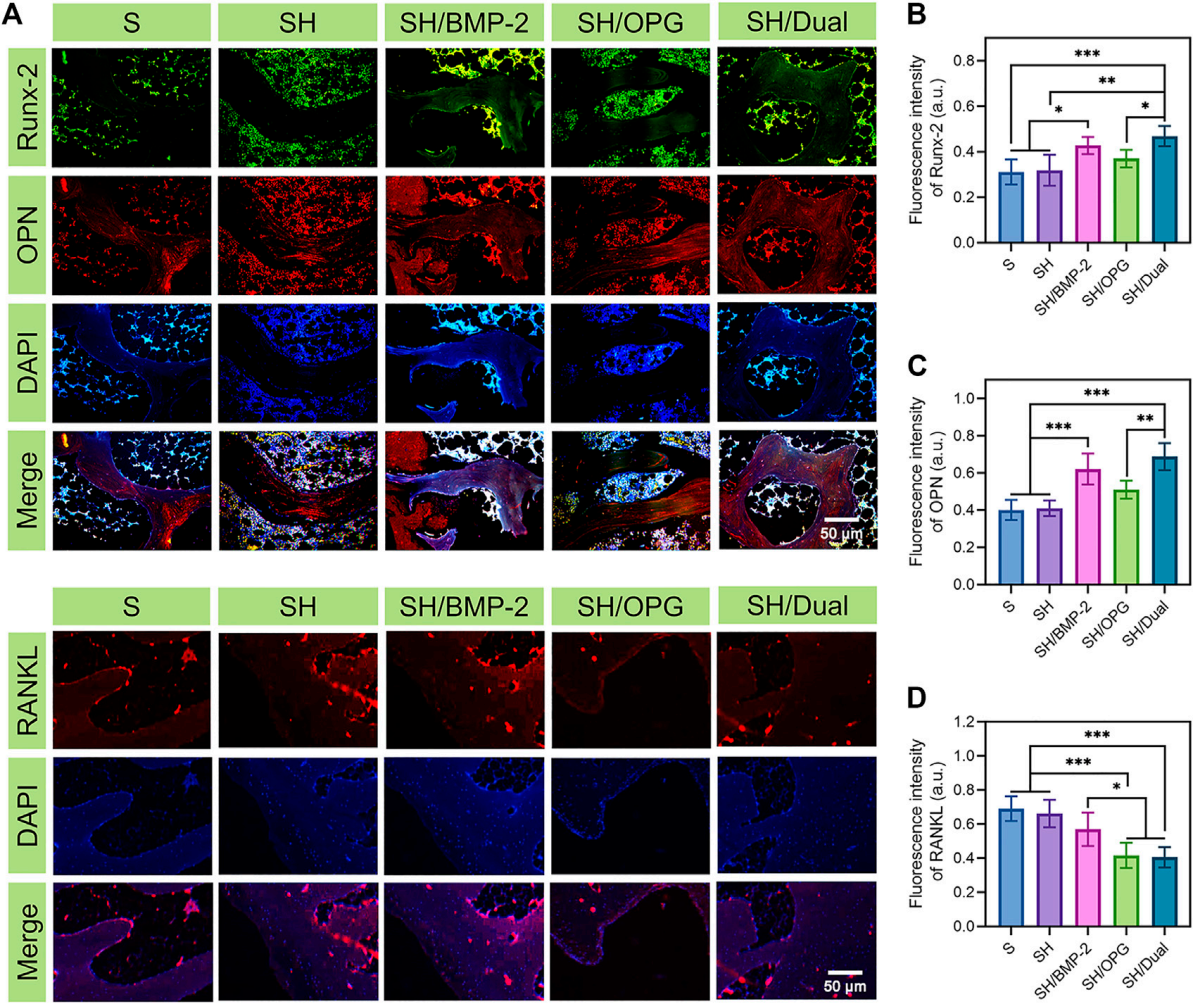
Immunocytochemical staining of the bone interface in each group. **(A)** The immunocytochemical images of osteogenic- and osteoclastic-related proteins (Runx-2, OPN, and RANKL) in each group. **(B–D)** The quantitative analyses of Runx-2, OPN, and RANKL expression. * indicates significant difference between groups, *p < 0.05, **p < 0.01, and ***p < 0.001.

To evaluate the expression of the bone formation and bone resorption-related markers, Runx-2, OPN, and RANKL were labeled *via* immunofluorescence staining at the bone interface after scaffolds were pushed out (Figure 5A). Figures 5B,C show that the expressions of Runx-2 and OPN were much higher in the SH/BMP-2 and SH/Dual groups than in the S, SH, and SH/OPG groups. As expected, the osteogenic-related proteins were higher in the SH/Dual groups compared with BMP-2 alone by using fluorescence intensity analyses. It is well known that the level of Runx-2 and OPN reflects a bone-regenerating environment (James et al., 2016; Wang et al., 2016). The fluorescence intensity analysis demonstrated that the existence of OPG reduced RANKL expression levels, thus inhibiting osteoclast activity. The cooperation of BMP-2 and OPG decreased RANKL to a higher degree compared with OPG loading alone (Figure 5D). This result may be because the cooperation of BMP-2 increased the regeneration of osteoblasts (Zheng et al., 2021). The activation of osteoblasts improved the osteogenic microenvironment in osteoporotic defects, and as a result, the osteoclast function was inhibited (Kim et al., 2021). The synergistic release of BMP-2 and OPG enhanced the osteogenic activity, inhibited the osteoclastic activity around the osteoporotic bone interface, and finally promoted the osseointegration of the 3D-printed composite scaffolds.

## Conclusion

To address the challenges of poor osseointegration in patients with osteoporosis after joint replacement, we developed a novel 3D-printed bioactive system that allowed for the sustained release of growth factors. In this study, 3D-printed Ti6Al4V porous scaffolds were filled with BMP-2- and OPG-loaded hydrogels. The superior efficacy of this therapy was systematically proved by its biological functions of maintaining osteoporosis-derived BMSC proliferation, viability, and differentiation *in vitro* and significantly improving bone generation and osseointegration. Therefore, this provides a new strategy for reducing postoperative complications and improving the outcome of joint replacement in patients with osteoporosis.

## Data Availability

The original contributions presented in the study are included in the article/Supplementary Material, further inquiries can be directed to the corresponding authors.
