# Supplementary material for: Incorporation of Bone Morphogenetic Protein-2 and Osteoprotegerin in 3D-Printed Ti6Al4V Scaffolds Enhances Osseointegration Under Osteoporotic Conditions
Source: Front Bioeng Biotechnol. 2021 Nov 4;9:754205. doi: 10.3389/fbioe.2021.754205 (PMC8600075; doi:10.3389/fbioe.2021.754205)
Supplement: Supplementary file 1 [file DataSheet1.PDF]

Supplementary Material

BMP-2 and OPG Incorporated 3D Printed Bioactive Interface Enhanced Osseointegration in Osteoporosis

Xianggang Wang[1,2, †], Zhengyan Li[3,4, †], Zhonghan Wang[1,2], He Liu[1,2], Yutao Cui[1,2],

Yuzhe Liu[1,2], Ming Ren[1,2], Hongsheng Zhan[3,4], Zuhao Li[1,2,*], Minfei Wu[1,2,*], and Jincheng Wang[1,2,*]

[1] Orthopaedic Medical Center, The Second Hospital of Jilin University, Changchun 130041, P. R. China.

[2] Orthopaedic Research Institute of Jilin Province, Changchun 130041, P. R. China

[3] Shi's Center of Orthopedics and Traumatology, Shuguang Hospital Affiliated to Shanghai University of TCM, Shanghai 201203, P. R. China.

[4] Institute of Traumatology, Shanghai Academy of TCM, Shanghai 201203, P. R. China.

*** Correspondence:**
Zuhao Li (lizuhao1992@163.com),

Minfei Wu (wuminfei100@163.com),

Jincheng Wang (wangjinc@jlu.edu.cn).

† Xianggang Wang and Zhengyan Li contributed equally to this work.

1. Supplementary Figures

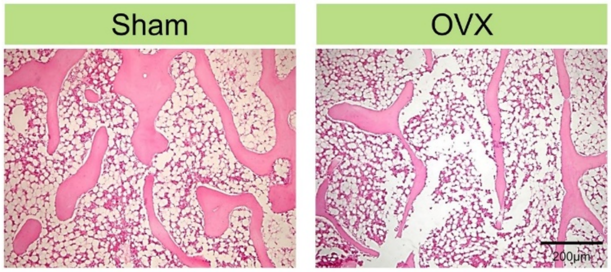

Figure. S1. H&E staining of the distal femur in sham and OVX groups at 10 months after OVX.

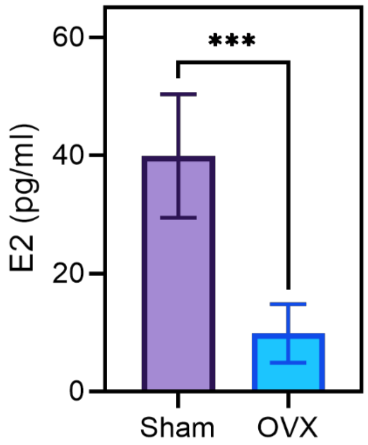

Figure. S2. Serum estrogen (E2) levels at 10 months after OVX (* indicates significant difference between groups, ***p < 0.001).

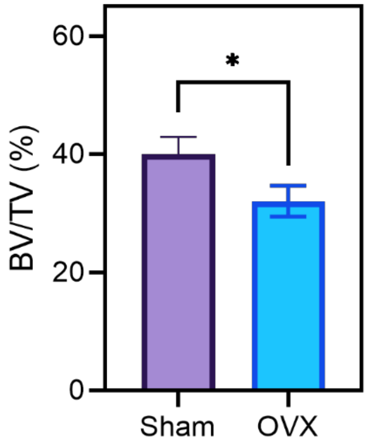

Figure. S3. Quantitative analysis of BV/TV in OVX and sham groups at 10 months after OVX (* indicates significant difference between groups, *$p < 0.05$).

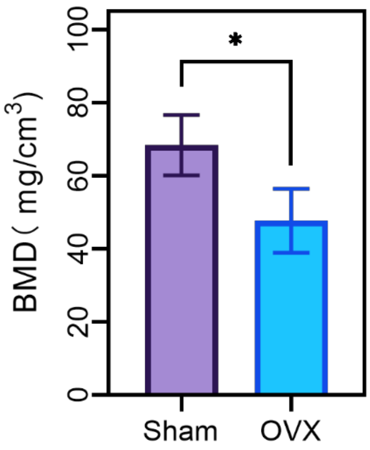

Figure. S4. Quantitative analysis of BMD values between rabbits in OVX and sham groups at 10 months after OVX. (* indicates significant difference between groups, *$p < 0.05$).

2. Supplementary Tables

Table S1. Primer sequences of genes

Gene subtype	Oligonucleotide Primers (5’-3’)
Runx-2	F: 5’-ACTACCAGCCACCGAGACCA-3’
	R: 5’-ACTGCTTGCAGCCTTAAATGACTCT-3’
OPN	F: 5’-GCTAAACCCTGACCCATCT-3’
	F: 5’-CGTCGGATTCATTGGAGT-3’
RANKL	F: 5’-GCCCTGTTCCTCTACTTCCG-3’
	R: 5’-CGGCTCTCAGGTGTTGTGAT-3’
GAPDH	F: 5’-GTATGATTCCACCCACGGCA-3’
	R: 5’-CCAGCATCACCCCACTTGAT-3’
